# Genetic variants in *RET*, *ARHGEF3* and *CTNNAL1*, and relevant interaction networks, contribute to the risk of Hirschsprung disease

**DOI:** 10.18632/aging.102891

**Published:** 2020-03-06

**Authors:** Yang Wang, Qian Jiang, Hao Cai, Ze Xu, Wenjie Wu, Beilin Gu, Long Li, Wei Cai

**Affiliations:** 1 Department of Pediatric Surgery, Xinhua Hospital, School of Medicine, Shanghai Jiao Tong University, Shanghai, China; 2 Department of Medical Genetics, Capital Institute of Pediatrics, Beijing, China; 3 Shanghai Key Laboratory of Pediatric Gastroenterology and Nutrition, Shanghai, China; 4 Shanghai Institute for Pediatric Research, Shanghai, China; 5 Department of General Surgery, Capital Institute of Pediatrics Affiliated Children's Hospital, Beijing, China

**Keywords:** *RET*, *ARHGEF3*, *CTNNAL1*, Hirschsprung disease, genetic interaction networks

## Abstract

Hirschsprung disease (HSCR), the most common enteric neuropathy, stands as a model for complex genetic disorders. It has recently been demonstrated that both *ARHGEF3* and *CTNNAL1* map to the *RET*-dependent HSCR susceptibility loci. We therefore sought to explore whether genetic variants within *RET*, *ARHGEF3* and *CTNNAL1*, and their genetic interaction networks are associated with HSCR. Taking advantage of a strategy that combined the MassArray system and gene-gene interaction analysis with case-control study, we interrogated 38 polymorphisms within *RET, ARHGEF3* and *CTNNAL1* in 1015 subjects (502 HSCR cases and 513 controls) of Han Chinese origin. There were statistically significant associations between 20 genetic variants in these three genes and HSCR. Haplotype analysis also revealed some significant global *P* values, i.e. *RET*_ rs2435357-rs752978-rs74400468-rs2435353-rs2075913-rs17028-rs2435355 (*P* = 3.79×10^-58^). Using the MDR and GeneMANIA platforms, we found strong genetic interactions among *RET*, *ARHGEF3*, and *CTNNAL1* and our previously studied *GAL*, *GAP43*, *NRSN1*, *PTCH1*, *GABRG2* and *RELN* genes. These results offer the first indication that genetic markers of *RET*, *ARHGEF3* and *CTNNAL1* and relevant genetic interaction networks confer the altered risk to HSCR in the Han Chinese population.

## INTRODUCTION

Hirschsprung disease (HSCR, MIM 142623), as a life-threatening genetic disorder, affects approximately 1/5000 live births around the world (2.8/10000 newborns in Asian population), which makes it the most frequent congenital disease of intestinal motility [1]. HSCR is a functional intestinal obstruction characterized by congenital absence of enteric neurons along a variable length of the bowel, and accordingly, it can be anatomically classified as short segment HSCR (S-HSCR), long segment HSCR (L-HSCR) and total colonic aganglionosis (TCA), and approximately 80% of HSCR cases belong to S-HSCR [2]. Of note, Hirschsprung disease has also been identified in elderly patients, including a 70-year-old male and a 67-year-old female [3, 4]. Additionally, gastrointestinal abnormalities were observed in patients with Parkinson's disease (PD) over 30 years ago, indicating that the enteric nervous system (ENS) might be involved in PD, and mouse models of PD pathogenesis also show varying degrees of enteric neuronal dysfunction [5].

The major causes of Hirschsprung disease can be attributed to genetic factors or gene-gene interaction networks, since HSCR presents crucial features of multifactorial genetic models, such as low sex-dependent penetrance, high sibling recurrence risk, high heritability and interfamilial variation [6]. Approximately 80% of HSCR cases are sporadic, and the rest are familial; moreover, HSCR is associated with other congenital malformations in approximately 30% of cases [1, 7]. Resembling other complex diseases, a single homozygous null mutation of HSCR susceptibility genes is not sufficient to lead to a serious aganglionosis phenotype in HSCR [8]. To date, genetic studies have revealed that at least 15 genes might be involved in HSCR development, yet these genes account for only ~ 30% of all HSCR cases [9], implying the involvement of more genes in HSCR susceptibility. Whole exome sequencing study has revealed some novel HSCR genes, including *DENND3*, *NCLN*, *NUP98* and *TBATA*, all of which might play a role in neuronal development [10]. More recently, new loci have been uncovered to be associated with Hirschsprung disease, such as *ALDH1A2*, *PLD1*, *CASQ2* and *CCT2* [11–13].

*RET* (ret proto-oncogene, located on chromosome 10q11.2), the major gene in HSCR, accounts for more than 80% of all known mutations [10, 14]. A recent genome-wide association study has revealed the key role of non-coding *RET* variants in HSCR [15]. Moreover, it has been indicated that in most HSCR cases, individuals with non-coding *RET* mutations also carry modifier loci, which contribute to HSCR presentation and phenotype severity, suggesting the potential interactions between *RET* and other genes involved in HSCR pathogenesis [16]. Taking advantage of the microarray technique and mouse model, Heanue et al. have demonstrated that both *Arhgef3* (Rho guanine nucleotide exchange factor (GEF) 3) and *Ctnnal1* (catenin (cadherin associated protein), alpha-like 1) have human homologues that map to previously identified HSCR susceptibility loci, i.e. the *RET*-dependent susceptibility loci (3p21 and 9q31), which makes them excellent candidate genes for Hirschsprung disease [17–19]. In addition, we wondered whether the interactions between *RET*, *ARHGEF3* and *CTNNAL1* contribute to the altered risk of HSCR since the joint gene-gene effects may have a significant impact on HSCR development [20]. It has recently been demonstrated that the interaction between *RET* and *PHOX2B* polymorphisms substantially affects the risk of Hirschsprung disease, suggesting that HSCR, as a multifactorial genetic disorder, requires the interactions of multiple unlinked genes to produce the phenotype [20]. In a recent study, we have shown that genetic markers within *GAL*, *GAP43* and *NRSN1* contribute to the altered HSCR susceptibility, and importantly, the interaction networks among *GAP43*, *NRSN1* and *PTCH1* confer an increased risk to Hirschsprung disease [21].

With all these lines of evidence, we tried to determine whether genetic variants of *RET, ARHGEF3* and *CTNNAL1* have an impact on the risk of Hirschsprung disease, and by recruiting 1015 subjects and 38 polymorphisms within these three genes, we further explored the potential interaction networks among *RET*, *ARHGEF3*, and *CTNNAL1* and our previously studied *GAL*, *GAP43*, *NRSN1*, *PTCH1*, *GABRG2* and *RELN* genes.

## RESULTS

### Allele and genotype distributions of the genetic variants in *RET*, *ARHGEF3*, and *CTNNAL1*

In the 1015 subjects, genotype distributions of all 38 polymorphisms showed no significant deviations from Hardy-Weinberg equilibrium in either HSCR cases or normal controls (*P*> 0.05). The allele and genotype frequencies of the 38 SNPs are listed in Tables 1–3. We found that there were significant associations between HSCR and 20 genetic variants, including 13 *RET* SNPs, 3 *ARHGEF3* SNPs, and 4 *CTNNAL1* SNPs. Of note, all 13 positive SNPs in *RET* survived the FDR correction in terms of both allele and genotype distributions (Table 1), and the findings observed in genotype distributions of *ARHGEF3* rs3732508 and in allele distributions of the 3 *ARHGEF3* SNPs and the 4 *CTNNAL1* SNPs remained significant after the FDR correction (Tables 2, 3). In addition, compared with previous findings regarding the 4 *RET* variants (rs2506030, rs7069590, rs2505998 and rs2435357), our present results have shown that the odds ratios have the same magnitudes, suggesting that the cases studied here are representative of Hirschsprung disease (Table 4). Regarding the 20 positive markers, we observed that certain alleles and genotypes presented markedly higher frequencies in the HSCR group than in the control group, such as the T allele and TT genotype of *RET* rs2435357, the A allele and AA genotype of *ARHGEF3* rs3732508, and the G allele and GG genotype of *CTNNAL1* rs4978379. Moreover, we also employed Plink software to perform the adjustment for age and gender factors, and all 20 positive polymorphisms survived the correction.

**Table 1:** Allele and genotype distributions of *RET* among HSCR patients and normal controls.

**SNP ID**	**Genotype frequency (%)**			H-W check *p* value*	*P* value*	**FDR adjusted**	**Allele frequency (%)**		**X^2^**	*P* value*	**FDR adjusted**	**Odds Ratio (95%CI)**
**rs2506030**	AA	AG	GG				A	G				
Case	15(3.0)	137(27.5)	346(69.5)	0.748	**9.90 x 10^-9^**	**1.16 x 10^-8^**	167(16.8)	829(83.2)	37.909	**7.64 x 10^-10^**	**8.91 x 10^-10^**	0.51 (0.41-0.63)
Control	42(8.2)	205(40.0)	265(51.8)	0.790			289(28.2)	735(71.8)				
**rs7069590**	CC	CT	TT				C	T				
Case	15(3.0)	134(26.7)	353(70.3)	0.600	**3.32 x 10^-10^**	**4.23 x 10^-10^**	164(16.3)	840(83.7)	44.086	**3.25 x 10^-11^**	**4.14 x 10^-11^**	0.49 (0.39-0.60)
Control	41(8.0)	212(41.3)	260(50.7)	0.808			294(28.7)	732(71.3)				
**rs2505998**	AA	AG	GG				A	G				
Case	313(62.4)	161(32.1)	28(5.6)	0.231	**7.86 x 10^-32^**	**5.50 x 10^-31^**	787(78.4)	217(21.6)	243.675	**6.22 x 10^-55^**	**4.35 x 10^-54^**	4.50 (3.70-5.46)
Control	101(19.7)	256(49.9)	156(30.4)	0.827			458(44.6)	568(55.4)				
**rs2435357**	CC	CT	TT				C	T				
Case	25(5.1)	146(29.7)	320(65.2)	0.124	**1.29 x 10^-52^**	**1.81 x 10^-51^**	196(20.0)	786(80.0)	269.208	**1.69 x 10^-60^**	**2.37 x 10^-59^**	0.20 (0.16-0.24)
Control	157(30.8)	252(49.5)	100(19.6)	0.950			566(55.6)	452(44.4)				
**rs752978**	CC	CT	TT				C	T				
Case	338(67.6)	140(28.0)	22(4.4)	0.131	**1.38 x 10^-30^**	**6.44 x 10^-30^**	816(81.6)	184(18.4)	143.721	**4.45 x 10^-33^**	**2.08 x 10^-32^**	3.35 (2.74-4.10)
Control	163(32.0)	255(50.0)	92(18.0)	0.655			581(57.0)	439(43.0)				
**rs74400468**	CC	CG	GG				C	G				
Case	3(0.6)	48(9.6)	449(89.8)	0.177	**5.87 x 10^-6^**	**6.32 x 10^-6^**	54(5.4)	946(94.6)	23.750	**1.12 x 10^-6^**	**1.21 x 10^-6^**	0.44 (0.32-0.62)
Control	7(1.4)	103(20.1)	402(78.5)	0.890			117(11.4)	907(88.6)				
rs3026737	CC	CT	TT				C	T				
Case	211(42.4)	234(47.0)	53(10.6)	0.316	0.502	> 0.05	656(65.9)	340(34.1)	0.016	0.899	> 0.05	1.01 (0.84-1.22)
Control	224(43.7)	225(43.9)	64(12.5)	0.522			673(65.6)	353(34.4)				
**rs1864402**	GG	GT	TT				G	T				
Case	267(53.7)	185(37.2)	45(9.1)	0.119	**2.80 x 10^-11^**	**4.36 x 10^-11^**	719(72.3)	275(27.7)	50.250	**1.41 x 10^-12^**	**1.97 x 10^-12^**	1.95 (1.62-2.35)
Control	169(33.1)	247(48.3)	95(18.6)	0.776			585(57.2)	437(42.8)				
**rs2075910**	AA	AG	GG				A	G				
Case	280(56.2)	178(35.7)	40(8.0)	0.124	**7.72 x 10^-24^**	**2.16 x 10^-23^**	738(74.1)	258(25.9)	116.026	**5.05 x 10^-27^**	**1.18 x 10^-26^**	2.76 (2.29-3.33)
Control	137(26.7)	248(48.3)	128(25.0)	0.457			522(50.9)	504(49.1)				
**rs2435353**	CC	CT	TT				C	T				
Case	386(77.7)	101(20.3)	10(2.0)	0.269	**3.57 x 10^-11^**	**5.00 x 10^-10^**	873(87.8)	121(12.2)	52.482	**4.52 x 10^-13^**	**7.03 x 10^-13^**	2.37 (1.87-3.00)
Control	298(58.2)	175(34.2)	39(7.6)	0.066			771(75.3)	253(24.7)				
**rs2075913**	AA	AT	TT				A	T				
Case	25(5.0)	148(29.5)	328(65.5)	0.126	**1.00 x 10^-23^**	**2.33 x 10^-23^**	198(19.8)	804(80.2)	116.103	**4.86 x 10^-27^**	**1.36 x 10^-26^**	0.34 (0.28-0.42)
Control	96(18.8)	237(46.3)	179(35.0)	0.265			429(41.9)	595(58.1)				
**rs17028**	CC	CT	TT				C	T				
Case	388(77.4)	104(20.8)	9(1.8)	0.511	**2.36 x 10^-11^**	**4.13 x 10^-11^**	880(87.8)	122(12.2)	53.124	**3.26 x 10^-13^**	**5.71 x 10^-13^**	2.37 (1.87-3.00)
Control	298(58.1)	176(34.3)	39(7.6)	0.073			772(75.2)	254(24.8)				
**rs2742240**	AA	AT	TT				A	T				
Case	38(7.6)	174(34.9)	287(57.5)	0.111	**3.97 x 10^-25^**	**1.39 x 10^-24^**	250(25.1)	748(74.9)	122.217	**2.24 x 10^-28^**	**7.84 x 10^-28^**	0.35 (0.29-0.42)
Control	125(24.5)	248(48.6)	137(26.9)	0.543			498(48.8)	522(51.2)				
**rs2435355**	CC	CT	TT				C	T				
Case	10(2.0)	103(20.5)	389(77.5)	0.306	**2.35 x 10^-11^**	**4.7 x 10^-11^**	123(12.3)	881(87.7)	53.188	**3.16 x 10^-13^**	**6.32 x 10^-13^**	0.42 (0.33-0.53)
Control	39(7.6)	177(34.5)	297(57.9)	0.084			255(24.9)	771(75.1)				

*Pearson's *p* value, FDR = false discovery rate, SNP = single nucleotide polymorphism, CI = confidence interval, HSCR = Hirschsprung disease.

**Table 2:** Allele and genotype distributions of *ARHGEF3* among HSCR patients and normal controls.

**SNP ID**	**Genotype frequency (%)**			H-W check *p* value*	*P* value*	**FDR adjusted**	**Allele frequency (%)**		**X2**	*P* value*	**FDR adjusted**	**Odds Ratio (95%CI)**
rs11717604	CC	CT	TT				C	T				
Case	219(43.7)	235(46.9)	47(9.4)	0.155	0.873	> 0.05	673(67.2)	329(32.8)	0.005	0.946	> 0.05	1.01(0.84-1.21)
Control	226(44.2)	233(45.6)	52(10.2)	0.476			685(67.0)	337(33.0)				
rs4681946	AA	AG	GG				A	G				
Case	212(42.5)	227(45.5)	60(12.0)	0.949	0.811	> 0.05	651(65.2)	347(34.8)	0.115	0.735	> 0.05	1.03(0.86-1.24)
Control	216(42.4)	226(44.3)	68(13.3)	0.467			658(64.5)	362(35.5)				
**rs11720618**	CC	CG	GG				C	G				
Case	32(6.4)	200(40.1)	267(53.5)	0.502	**0.012**	> 0.05	264(26.5)	734(73.5)	8.358	**0.004**	**0.025**	1.35(1.10-1.66)
Control	18(3.5)	178(34.9)	314(61.6)	0.235			214(21.0)	806(79.0)				
rs13070800	CC	CT	TT				C	T				
Case	84(16.8)	236(47.3)	179(35.9)	0.679	0.889	> 0.05	404(40.5)	594(59.5)	0.211	0.646	> 0.05	0.96(0.80-1.15)
Control	89(17.4)	246(48.1)	176(34.4)	0.849			424(41.5)	598(58.5)				
**rs11925835**	CC	CT	TT				C	T				
Case	61(12.2)	223(44.6)	216(43.2)	0.768	**0.039**	> 0.05	345(34.5)	655(65.5)	6.553	**0.010**	**0.045**	1.28(1.06-1.54)
Control	44(8.6)	211(41.2)	257(50.2)	0.941			299(29.2)	725(70.8)				
**rs3732508**	AA	AG	GG				A	G				
Case	28(5.6)	201(40.4)	269(54.0)	0.227	**0.003**	**0.033**	257(25.8)	739(74.2)	10.315	**0.001**	**0.017**	1.41(1.14-1.74)
Control	22(4.3)	158(31.0)	330(64.7)	0.577			202(19.8)	818(80.2)				
rs9882898	AA	AG	GG				A	G				
Case	244(49.0)	211(42.4)	43(8.6)	0.784	0.189	> 0.05	699(70.2)	297(29.8)	3.262	0.071	> 0.05	1.19(0.99-1.43)
Control	226(44.2)	227(44.4)	58(11.4)	0.930			679(66.4)	343(33.6)				
rs3732509	CC	CG	GG				C	G				
Case	33(6.6)	197(39.5)	269(53.9)	0.703	0.529	> 0.05	263(26.4)	735(73.6)	1.234	0.267	> 0.05	0.90(0.74-1.09)
Control	41(8.0)	211(41.1)	261(50.9)	0.856			293(28.6)	733(71.4)				
rs3772219	AA	AC	CC				A	C				
Case	193(38.5)	240(47.9)	68(13.6)	0.627	0.934	> 0.05	626(62.5)	376(37.5)	0.046	0.830	> 0.05	1.02(0.85-1.22)
Control	192(37.5)	251(49.0)	69(13.5)	0.359			635(62.0)	389(38.0)				
rs3732511	CC	CG	GG				C	G				
Case	222(44.5)	220(44.1)	57(11.4)	0.823	0.988	> 0.05	664(66.5)	334(33.5)	0.012	0.912	> 0.05	0.99(0.82-1.19)
Control	229(44.6)	227(44.2)	57(11.1)	0.947			685(66.8)	341(33.2)				
rs1009119	CC	CT	TT				C	T				
Case	57(11.4)	219(43.9)	223(44.7)	0.771	0.985	> 0.05	333(33.4)	665(66.6)	0.004	0.950	> 0.05	1.01(0.84-1.21)
Control	57(11.1)	227(44.2)	229(44.6)	0.947			341(33.2)	685(66.8)				
rs6978	AA	AG	GG				A	G				
Case	56(11.2)	217(43.6)	225(45.2)	0.736	0.978	> 0.05	329(33.0)	667(67.0)	0.013	0.910	> 0.05	0.99(0.82-1.19)
Control	57(11.2)	226(44.2)	228(44.6)	0.929			340(33.3)	682(66.7)				
rs808	AA	AG	GG				A	G				
Case	309(61.7)	171(34.1)	21(4.2)	0.662	0.857	> 0.05	789(78.7)	213(21.3)	0.050	0.823	> 0.05	1.02(0.83-1.27)
Control	314(61.6)	171(33.5)	25(4.9)	0.782			799(78.3)	221(21.7)				

*Pearson's *p* value, FDR = false discovery rate, SNP = single nucleotide polymorphism, CI = confidence interval, HSCR = Hirschsprung disease.

**Table 3:** Allele and genotype distributions of *CTNNAL1* among HSCR patients and normal controls.

**SNP ID**	**Genotype frequency (%)**			H-W check *p* value*	*P* value*	**FDR adjusted**	**Allele frequency (%)**		**X^2^**	*P* value*	**FDR adjusted**	**Odds Ratio (95%CI)**
rs10816766	CC	CT	TT				C	T				
Case	32(6.4)	164(32.7)	305(60.9)	0.123	0.249	> 0.05	228(22.8)	774 (77.2)	0.484	0.487	> 0.05	1.08(0.87-1.33)
Control	21(4.1)	177(34.7)	312(61.2)	0.510			219(21.5)	801(78.5)				
**rs10979650**	AA	AG	GG				A	G				
Case	23(4.6)	146(29.3)	330(66.1)	0.192	**0.017**	> 0.05	192(19.2)	806(80.8)	7.241	**0.007**	**0.039**	1.38(1.09-1.74)
Control	10(2.0)	131(25.6)	371(72.5)	0.690			151(14.7)	873(85.3)				
**rs4978766**	AA	AG	GG				A	G				
Case	333(66.3)	147(29.3)	22(4.4)	0.267	**0.025**	> 0.05	813(81.0)	191(19.0)	6.617	**0.010**	**0.028**	0.73(0.58-0.93)
Control	371(72.5)	131(25.6)	10(2.0)	0.690			873(85.3)	151(14.7)				
**rs4978379**	CC	CG	GG				C	G				
Case	327(65.5)	146(29.3)	26(5.2)	0.073	**0.005**	> 0.05	800(80.2)	198(19.8)	9.437	**0.002**	**0.023**	0.70(0.55-0.88)
Control	371(72.6)	130(25.4)	10(2.0)	0.722			872(85.3)	150(14.7)				
rs2282206	AA	AG	GG				A	G				
Case	144(28.8)	248(49.6)	108(21.6)	0.950	0.768	> 0.05	536(53.6)	464(46.4)	0.104	0.748	> 0.05	0.97(0.82-1.16)
Control	156(30.6)	242(47.5)	112(22.0)	0.322			554(54.3)	466(45.7)				
rs838816	CC	CT	TT				C	T				
Case	113(22.6)	251(50.2)	136(27.2)	0.891	0.600	> 0.05	477(47.7)	523(52.3)	0.140	0.708	> 0.05	1.03(0.87-1.23)
Control	119(23.3)	241(47.2)	151(29.5)	0.231			479(46.9)	543(53.1)				
rs838817	CC	CT	TT				C	T				
Case	141(28.1)	249(49.7)	111(22.2)	0.957	0.614	> 0.05	531(53.0)	471(47.0)	0.250	0.617	> 0.05	0.96(0.80-1.14)
Control	157(30.7)	240(46.9)	115(22.5)	0.204			554(54.1)	470(45.9)				
**rs7021366**	CC	CG	GG				C	G				
Case	195(39.0)	235(47.0)	70(14.0)	0.952	**0.020**	> 0.05	625(62.5)	375(37.5)	7.169	**0.007**	**0.027**	0.78(0.65-0.95)
Control	244(47.7)	210(41.0)	58(11.3)	0.214			698(68.2)	326(31.8)				
rs7027874	AA	AG	GG				A	G				
Case	143(28.7)	243(48.8)	112(22.5)	0.651	0.784	> 0.05	529(53.1)	467(46.9)	0.443	0.505	> 0.05	0.94(0.79-1.12)
Control	157(30.7)	245(047.9)	110(21.5)	0.431			559(54.6)	465(45.4)				
rs2289481	CC	CG	GG				C	G				
Case	111(22.2)	246(49.3)	142(28.5)	0.820	0.711	> 0.05	468(46.9)	530(53.1)	0.386	0.534	> 0.05	1.06(0.89-1.26)
Control	112(21.8)	243(47.4)	158(30.8)	0.309			467(45.5)	559(54.5)				
rs2289480	AA	AC	CC				A	C				
Case	141(28.3)	246(49.3)	112(22.4)	0.811	0.673	> 0.05	528(52.9)	470(47.1)	0.506	0.477	> 0.05	0.94(0.79-1.12)
Control	158(30.8)	243(47.4)	112(21.8)	0.309			559(54.5)	467(45.5)				

*Pearson's *p* value, FDR = false discovery rate, SNP = single nucleotide polymorphism, CI = confidence interval, HSCR = Hirschsprung disease.

**Table 4:** The comparison of 4 HSCR-associated genetic variants in *RET* between previous findings and the present results.

**SNP ID**	**Groups**	**Risk/Non-risk allele**	**Risk allele frequency (Case/Control)**	**Odds Ratio (95%CI)**	*P* value*
**rs2506030**	Previous findings**	G/A	0.54/0.40	1.7(1.4-2.2)	2.2 x 10^-6^
	The present results		**0.83/0.72**	**2.0(1.6-2.4)**	**8.9 x 10^-10^**
**rs7069590**	Previous findings**	T/C	0.84/0.74	1.8(1.3-2.5)	9.7 x 10^-5^
	The present results		**0.84/0.71**	**2.1(1.7-2.5)**	**4.1 x 10^-11^**
**rs2505998**	Previous findings**	A/G	0.64/0.22	4.2(3.2-5.3)	1.1 x 10^-28^
	The present results		**0.78/0.45**	**4.5(3.7-5.5)**	**4.4 x 10^-54^**
**rs2435357**	Previous findings**	T/C	0.59/0.23	4.8(3.8-6.1)	6.0 x 10^-40^
	The present results		**0.80/0.44**	**5.0(4.1-6.1)**	**2.4 x 10^-59^**

*Pearson's *p* value, **European studies [14, 26], FDR = false discovery rate, SNP = single nucleotide polymorphism, CI = confidence interval, HSCR = Hirschsprung disease.

### Linkage disequilibrium analysis

**Figure 1:**
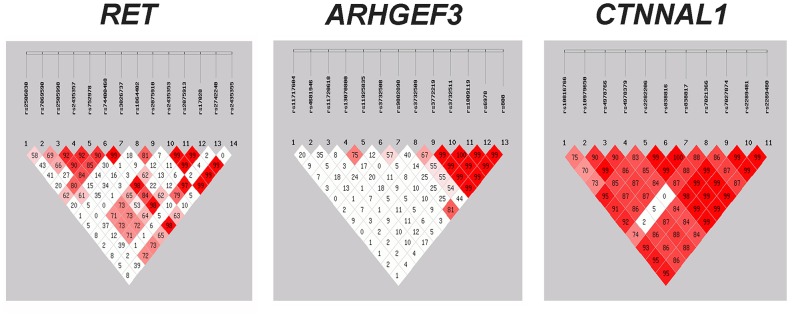
**Assessment of linkage disequilibrium (LD) between the genetic variants within *RET, ARHGEF3* and *CTNNAL1*.** The matrices represent the *D'* value between the SNP pairs. Red matrices denote *D'* > 70%. SNP = single nucleotide polymorphism.

We further conducted linkage disequilibrium (LD) analyses of genetic markers within the *RET*, *ARHGEF*, and *CTNNAL1* genes since LD makes tightly linked SNPs markedly correlated, leading to cost savings for association studies [22]. Figure 1 presents LD results for each pair of genetic variants in the HSCR group and control group. Strong LD (*D'* > 0.7) was found in multiple groups of markers, such as *RET*_rs2435353-rs2075913-rs17028, *ARHGEF3*_rs11720618-rs11925835-rs3732508 rs3732511-rs1009119-rs6978, *CTNNAL1*_rs10979650-rs4978766-rs4978379-rs838816-rs7021366 and etc. We therefore investigated the haplotype distributions for these SNPs in the later analysis.

### Haplotype analysis and power calculations

Since haplotype constructed from genetic markers with strong LD (*D'* > 0.7) will markedly increase the statistical power for association with the disease, we then carried out haplotype analyses of SNPs in these three genes. Moreover, haplotypes were omitted from the analysis if the estimated haplotype probabilities were less than 3% in either the HSCR or control group. Only haplotypes with strong LD were selected for presentation (Supplementary Table 1). In the present study, we observed that multiple haplotypes were significantly associated with HSCR. For the 38 genetic variants within these three genes, haplotype analysis also unraveled some significant global *P* values (Table 5). For each gene, the most significant haplotypes included *RET*_ rs2435357-rs752978-rs74400468-rs2435353-rs2075913-rs17028-rs2435355 (*P* = 3.79×10^-58^), *ARHGEF3*_rs11720618-rs11925835-rs3732508-rs3772219-rs3732511-rs1009119-rs6978-rs8 08 (*P* = 2.99×10^-8^), and *CTNNAL1*_rs10979650-rs4978 766-rs4978379-rs838816-rs7021366 (*P* = 5.05×10^-5^). Importantly, we found that the combinations of the *RET* risk haplotype reported previously [23] and the risk alleles of *ARHGEF3* and/or *CTNNAL1* showed increased odds ratios compared to those consisting of only the *RET* risk haplotype and the non-risk alleles of *ARHGEF3* and/or *CTNNAL1* (Supplementary Table 2). By using the G*Power 3 program, we demonstrated that our sample size had > 90% power to detect a significant association (α < 0.05) for genetic markers when an effect size index of 0.22 (corresponding to a "weak" gene effect) was adopted.

**Table 5:** Global p values of estimated haplotypes.

**Gene ID**	**Haplotype**	Global *p* value*
*RET*	rs2435353-rs2075913-rs17028	**3.66 x 10^-26^**
	rs74400468-rs2435353-rs2075913-rs17028-rs2435355	**5.40 x 10^-28^**
	rs1864402-rs2075910-rs2742240	**2.03 x 10^-26^**
	rs2435357-rs752978-rs74400468	**5.56 x 10^-56^**
	rs2505998-rs2435357-rs752978-rs74400468-rs2435353-rs2075913-rs17028-rs2435355	**1.46 x 10^-57^**
	rs2435357-rs752978-rs74400468-rs2435353-rs2075913-rs17028-rs2435355	**3.79 x 10^-58^**
*ARHGEF3*	rs11720618-rs11925835-rs3732508-rs9882898-rs3732509-rs3772219-rs3732511-rs100911-rs6978-rs808	**2.95 x 10^-6^**
	rs11720618-rs11925835-rs3732508	**5.38 x 10^-4^**
	rs11720618-rs11925835-rs3732508-rs3732511-rs1009119-rs6978	**1.67 x 10^-5^**
	rs11720618-rs11925835-rs3732508-rs3772219-rs3732511-rs1009119-rs6978-rs808	**2.99 x 10^-8^**
*CTNNAL1*	rs10816766-rs10979650-rs4978766-rs4978379-rs2282206-rs838816-rs838817-rs7021366-rs7027874-rs2289481-rs2289480	0.236
	rs10979650-rs4978766-rs4978379	0.063
	rs10979650-rs4978766-rs4978379-rs7021366	0.050
	rs10979650-rs4978766-rs4978379-rs7021366-rs7027874-rs2289481-rs2289480	0.096
	rs10979650-rs4978766-rs4978379-rs838816-rs7021366	**5.05 x 10^-5^**
	rs10979650-rs4978766-rs4978379-rs7021366-rs7027874	0.105
	rs10979650-rs4978766-rs4978379-rs7021366-rs2289481	0.112
	rs10816766-rs10979650-rs4978766-rs4978379-rs7021366	0.114

*Pearson's *p* value, statistical significance set at *p*<0.05, SNP = single nucleotide polymorphism.

### Genetic interaction network analysis

**Figure 2:**
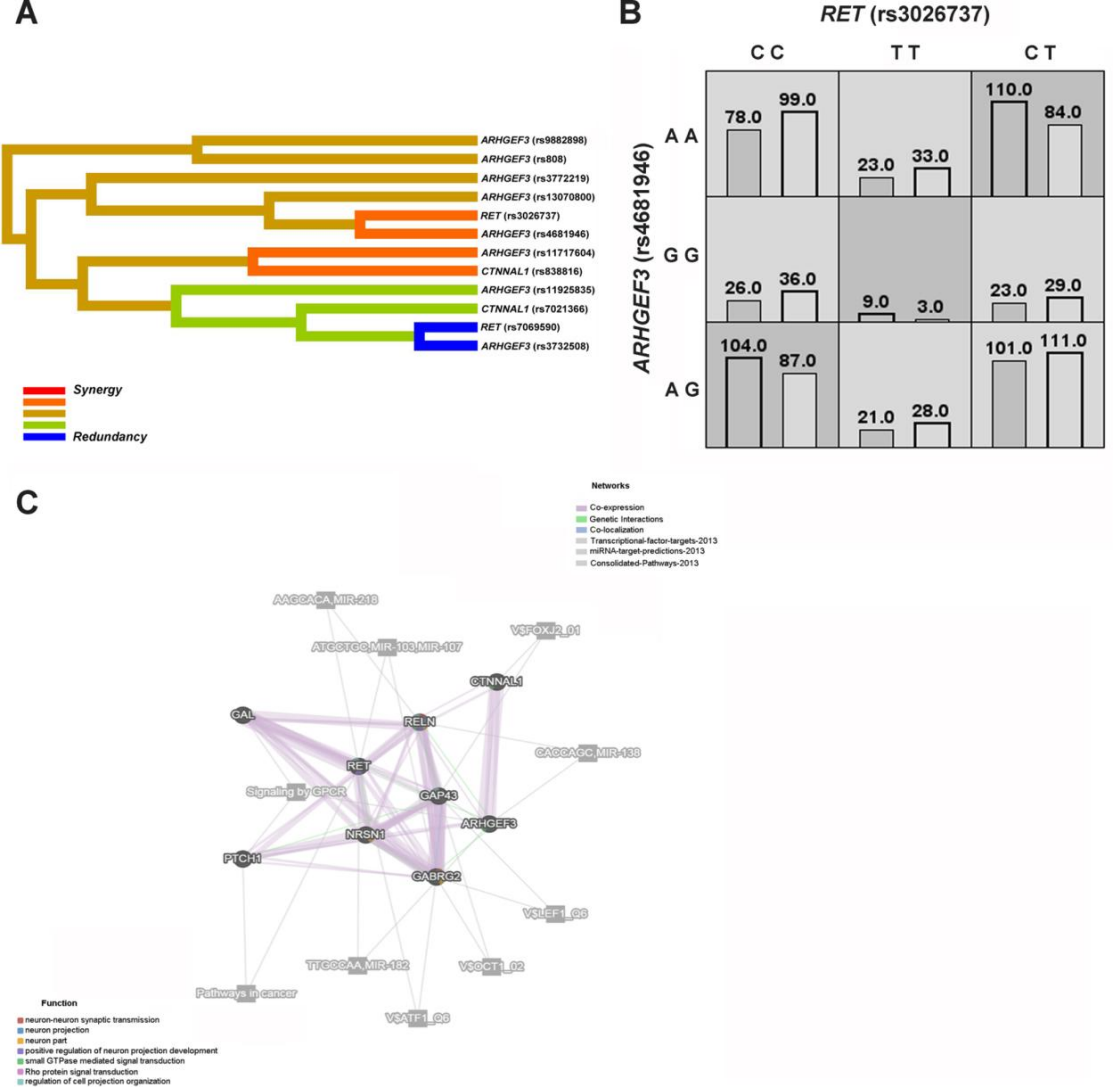
**HSCR-related genetic interaction networks among *RET, ARHGEF3*, *CTNNAL1* and our previously studied *GAL*, *GAP43*, *NRSN1*, *PTCH1*, *GABRG2* and *RELN* gene.** (**A**) The interaction dendrogram derived from MDR (Multifactor dimensionality reduction). Short connections among nodes represent stronger synergistic (red and orange) or redundant (green and blue) interactions. (**B**) In the two-factor best model, multilocus genotype combinations are linked to the altered HSCR risk. Darker-shaded cells represent higher risk combinations compared with lighter-shaded cells. Each cell denotes counts of HSCR subjects on left and controls on right. (**C**) The genetic interaction networks derived from GeneMANIA. The 9 HSCR-associated genes are linked to each other by the functional association networks in the GeneMANIA system.

The multifactor dimensionality reduction (MDR) strategy was then recruited to assess the potential gene-gene interactions among *RET*, *ARHGEF3* and *CTNNAL1* (Figure 2A, 2B). Of note, the MDR analysis showed the best interaction models regarding HSCR risk, and accordingly, *RET* (rs7069590) was the best single factor model (Testing accuracy = 0.6337; Cross-validation consistency = 10/10) (Table 6). Moreover, the best two-factor model consisted of *RET* (rs3026737) and *ARHGEF3* (rs4681946), and specifically, certain genotype combinations, such as CT (rs3026737)-AA (rs4681946), might lead to an increased risk in HSCR (Figure 2B). On the other hand, *RET* (rs7069590), *ARHGEF3* (rs11717604), *ARHGEF3* (rs3732508), and *CTNNAL1* (rs7021366) constituted the best four-factor model (Testing accuracy = 0.5941; Cross-validation consistency = 10/10).

**Table 6:** Gene-gene interaction models for SNPs in HSCR risk by MDR analysis.

**Number of factors**	Best model*	**Training accuracy**	**Testing accuracy**	**CVC**	**X^2^**	***P* value**	**Odds Ratio (95%CI)**
1	*RET*(rs7069590)	0.593	0.6337	10/10	33.705	<0.0001	2.22(1.69-2.91)
2	*RET*(rs3026737)-*ARHGEF3*(rs4681946)	0.5569	0.5743	10/10	11.830	0.0006	1.59(1.22-2.08)
3	*RET*(rs3026737)-*RET*(rs7069590)-*ARHGEF3*(rs11925835)	0.6193	0.6238	10/10	55.266	<0.0001	2.80(2.13-3.69)
4	*RET*(rs7069590)-*ARHGEF3*(rs11717604)-*ARHGEF3*(rs3732508)-*CTNNAL1*(rs7021366)	0.6477	0.5941	10/10	79.921	<0.0001	3.41(2.59-4.47)

*The best model was referred to as the one with the maximum testing accuracy and maximum cross-validation consistency (CVC); MDR = multifactor dimensionality reduction, CI = confidence interval, HSCR = Hirschsprung disease.

We also used GeneMANIA, a flexible user-friendly database, to explore the functional association networks among *RET*, *ARHGEF3*, and *CTNNAL1* and our previously studied *GAL*, *GAP43*, *NRSN1*, *PTCH1*, *GABRG2* and *RELN* genes [21]. As shown in Figure 2C, gene function prediction indicated that *RET*, *ARHGEF3*, and *CTNNAL1* might be involved in the positive regulation of neuron projection development, regulation of cell projection organization, and small GTPase-mediated signal transduction. These nine genes interacted with each other mainly through co-expression and/or genetic interactions, and some of them might contribute to the same pathways, such as signaling by GPCR.

## DISCUSSION

HSCR is a congenital complex disorder caused by a deficit in the migration process of enteric neural crest cells (ENCCs). To date, more than 15 genes have been linked to the development of Hirschsprung disease, and yet, the landscape of genetic networks regarding HSCR risk has not been fully characterized. Here, we carried out linkage disequilibrium analyses of 38 genetic markers within the *RET*, *ARHGEF*, and *CTNNAL1* genes in 502 HSCR cases and 513 normal controls and identified significant associations of these three genes with altered HSCR susceptibility. Moreover, our present work for the first time unraveled that the interaction networks among *RET*, *ARHGEF3*, and *CTNNAL1* and our previously studied *GAL*, *GAP43*, *NRSN1*, *PTCH1*, *GABRG2* and *RELN* genes might contribute to an increased risk of HSCR.

Recent genetics studies have mainly focused on the major HSCR gene, *RET*, which contains at least 80% of all known HSCR-causing mutations [24]. Our present results showed that 13 out of 14 genetic variants within *RET* were significantly linked to the altered HSCR susceptibility. Of note, certain alleles and/or genotypes of these 13 positive SNPs might be risk factors for HSCR, such as the A allele and AA genotype of rs2505998, the T allele and TT genotype of rs2435357 and the C allele and CC genotype of rs752978, while the others might be protective factors against HSCR, such as the T allele and TT genotype of rs17028 and the C allele and CC genotype of rs2435355. Additionally, the 3 positive markers (rs17028, rs2742240 and rs2435355) may further affect the regulatory mechanisms of gene expression since all of them are located in the 3' UTR region [25]. Regarding the 4 SNPs (rs2506030, rs7069590, rs2505998 and rs2435357) that have been reported in Hirschsprung disease [14, 26], our findings also supported that these 4 markers contribute to the altered HSCR risk.

We then tried to interrogate the relationship between *ARHGEF3*, *CTNNAL1* and HSCR, since both of them were indicated as excellent candidate genes for HSCR [17]. Our current work presented strong associations of *ARHGEF3* and *CTNNAL1* with Hirschsprung disease. As the positive variant rs3732508 in *ARHGEF3* is synonymous, it may not change the amino acid directly, and yet, based on recent studies, synonymous SNPs can affect mRNA splicing, stability and structure as well as protein folding, which may cause changes in protein function and cellular response to therapeutic targets [27]. In humans, ARHGEF3 belongs to the family of RhoGEFs (Rho guanine nucleotide exchange factor), which promote GDP to GTP exchange [28]. A recent study showed that knockdown of *ARHGEF3* markedly inhibits NPC (nasopharyngeal carcinoma) cell growth and migration [29]. On the other hand, rs7021366_*CTNNAL1*, the positive marker, is a missense mutation, which results in a change in the amino acid and thus may substantially have an impact on the function of proteins. CTNNAL1 (α-Catulin) is a cytoplasmic molecule that integrates the crosstalk between nuclear factor-kappa B and Rho signaling pathways, and attenuation of α-Catulin *in vitro* blocked cell migration and invasion induced by other proteins [30, 31]. Regarding *ARHGEF3* and *CTNNAL1*, our work also revealed some potential risk factors for HSCR, such as the C allele and CC genotype of *ARHGEF3* rs11720618, the A allele and AA genotype of *ARHGEF3* rs3732508, and the G allele and GG genotype of *CTNNAL1* rs7021366, all of which might confer an increased risk of HSCR. Importantly, additional replication studies recruiting larger Asian and non-Asian samples and more markers will certainly be needed in the future.

On the other hand, our current findings also revealed some significant haplotypes, which are relevant to HSCR susceptibility (Table 5 and Supplementary Table 1). Compared with individual SNP analysis, haplotype analysis might increase the power to detect disease-causing loci [32], and in regards to the most significant haplotypes for each gene, some showed markedly higher frequencies in the HSCR group than in the control group, e.g., *RET*_A-T-C-G-C-T-C-T (rs2505998-rs2435357-rs752978-rs74400468-rs243535 3-rs2075913-rs17028-rs2435355, *P* = 4.69×10^-52^, OR = 4.83, 95% CI 3.92-5.96) and *ARHGEF3*_G-C-A-A-C-T-G-A (rs11720618-rs11925835-rs3732508-rs3772219-rs3732511-rs1009119-rs6978-rs808, *P* = 1.43×10^-4^, OR = 3.78, 95% CI 1.82-7.86), both of which are the potential risk factors for HSCR, whereas *CTNNAL1*_G-A-C-C-C (rs10979650-rs4978766-rs4978379-rs838816-rs7021366, *P* = 9.04×10^-6^, OR = 0.18, 95% CI 0.08-0.42) exerts a protective effect against HSCR.

Interestingly, both *ARHGEF3* and *CTNNAL1* map to the RET-dependent HSCR susceptibility loci identified at 3p21 and 9q31, respectively [18, 19]. Since ARHGEF3 encodes a RhoGEF and CTNNAL1 can interact with RhoGEFs, it has been indicated that the two genes potentially interact in the modulation of cell migration [17, 33]. We thus enrolled the multifactor dimensionality reduction (MDR) strategy to explore the gene-gene interactions among *RET*, *ARHGEF3* and *CTNNAL1* (Figure 2A, 2B), and interrogated the best interaction model with maximum testing accuracy and maximum cross-validation consistency (CVC) among these three genes. Of note, MDR is a nonparametric model-free method that does not require particular inheritance model to detect gene-gene interactions without main gene effects in case-control studies of complex diseases [34]. In our MDR analysis, the best interaction models also contained certain positive genetic variants associated with HSCR, including *RET*_rs7069590, *ARHGEF3*_rs11925835, *ARHGEF3*_rs3732508 and *CTNNAL1*_rs7021366 (Table 6). Interestingly, the best four-factor model, *RET* (rs7069590)-*ARHGEF3* (rs11717604)-*ARHGEF3* (rs3732508)-*CTNNAL1* (rs7021366), showed the most significant OR compared to other models, further supporting that a multifactor model might play a major role in HSCR susceptibility. Since 2 positive cSNPs (*ARHGEF3*_rs3732508 and *CTNNAL1*_rs7021366) were also involved in this best four-factor model, the interaction between them might have a functional impact on HSCR pathogenesis.

Taking advantage of the GeneMANIA platform, we then explored functionally related gene-gene interaction networks among *RET*, *ARHGEF3*, and *CTNNAL1*, and our previously studied *GAL*, *GAP43*, *NRSN1*, *PTCH1*, *GABRG2* and *RELN* genes [21]. These HSCR-associated genes were linked to each other through co-expression, genetic interactions, co-localization, or they might be involved in the same pathways or might be targeted by the same microRNAs (Figure 2C). Moreover, *RET*, *ARHGEF3* and *CTNNAL1* might functionally contribute to the regulation of cell projection organization, small GTPase-mediated signal transduction and positive regulation of neuron projection development, and thus they might be involved in HSCR etiology, as HSCR is due to a deficit in the development of the enteric nervous system.

Taken together, our present data show that genetic variants and haplotypes in *RET*, *ARHGEF3* and *CTNNAL1* confer an altered risk to Hirschsprung disease in the Han Chinese population. To the best of our knowledge, this study offers the first indication that the gene-gene interactions among *RET*, *ARHGEF3* and *CTNNAL1* contribute to an increased risk of HSCR. Moreover, we have also unraveled the potential interaction networks consisting of 9 HSCR-related genes, which might be involved in HSCR susceptibility. Future research is required to fully understand the complexity of genetic interaction networks involved in HSCR risk and address the genetic basis of Hirschsprung disease.

## MATERIALS AND METHODS

### Study subjects

In the present study, we recruited 1015 subjects, including 502 sporadic HSCR cases (383 males and 119 females, age 1.34 ± 2.12 years) and 513 normal controls (310 males and 203 females, age 2.70 ± 3.13 years). All the participants were of Han Chinese origin and were enrolled from biologically unrelated residents. The 502 cases in the study had the diagnosis of HSCR based on histological examination of surgical or biopsy resection material, including 369 S-HSCR (short segment HSCR), 74 L-HSCR (long segment HSCR) and 59 TCA (total colonic aganglionosis). Controls were randomly recruited from the general population with no history of chronic constipation. We received approval for the study from Xinhua Hospital and Capital Institute of Pediatrics and obtained written informed consent from participants or their parents after the procedure had been fully explained. DNA was extracted using the QIAamp DNA Blood Midi Kit (Qiagen, Valencia, CA).

### SNP selection and genotyping

**Figure 3:**
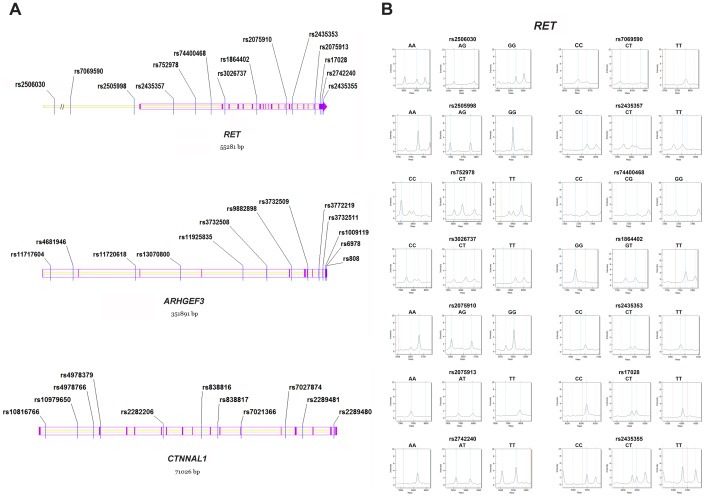
**Distribution and representative mass spectra of the genetic markers in the study.** (**A**) The 38 polymorphisms within *RET, ARHGEF3* and *CTNNAL1*. Blue lines denote the studied SNPs; Purple lines and arrows represent the exons in the genomic region; (**B**) Representative mass spectra of the 14 genetic variants within *RET*. Blue dotted lines denote the presence of the studied alleles; Red dotted lines indicate no allele detected; Grey dotted lines represent the unrelated peaks. SNP = single nucleotide polymorphism.

The tagSNP selection was conducted using the Genome Variation Server (http://gvs.gs.washington.edu/GVS150/) with MAF (minor allele frequency) ≥ 0.15 and r^2^ ≥ 0.8 according to the HapMap HCB (Han Chinese in Beijing) database. We thus enrolled 38 SNPs in the study, including 34 tagSNPs and 4 SNPs (*RET*: rs2506030, rs7069590, rs2505998 and rs2435357) that have been previously reported [14, 26]. All 38 SNPs consisted of 6 3' UTR SNPs (*RET*: rs17028, rs2742240 and rs2435355; *ARHGEF3*: rs1009119, rs6978 and rs808), 4 cSNPs (coding SNPs) (*ARHGEF3*: rs3732508, rs3772219 and rs3732511; *CTNNAL1*: rs7021366), and 28 intronic SNPs (Figure 3A). Genotyping was performed using the MassARRAY iPLEX Gold system (Sequenom, San Diego, CA), and Figure 3B presents representative mass spectra of the 14 genetic variants in the *RET* gene. Additionally, we recruited multiple criteria for genotyping quality control, similar to previous study [21].

### SNP-SNP interaction network analysis

In our present study, multifactor dimensionality reduction (MDR) analysis was employed to investigate the SNP-SNP interaction networks in regard to Hirschsprung disease. MDR software (version 3.0.2) was thus used to carry out the MDR analysis, and the risk factors were explored in the best model, which maximized both testing accuracy and cross-validation consistency (CVC) [35]. Specifically, MDR takes advantage of cross-validation by dividing the data into a training dataset (i.e. 9/10 of the data) and a testing dataset (i.e. the remaining 1/10 of the data) to derive estimates of cross-validation consistency and testing accuracy [35]. Moreover, GeneMANIA, a flexible user-friendly database, was used to further interrogate the gene-gene interaction networks and to perform a function prediction, and specifically, the interaction networks derived from GeneMANIA are based on multiple datasets, such as co-expression, genetic interactions and consolidated pathways datasets [36].

### Statistical analysis

SHEsis (http://analysis.bio-x.cn/myAnalysis.php) was used to assess Hardy-Weinberg equilibrium (HWE), odds ratio (OR), 95% confidence interval (CI), allelic and genotypic association, and to study linkage disequilibrium (LD), allelic and haplotype distribution [37]. The false discovery rate (FDR) controlling procedure was used to correct the *P* values of genetic analysis [38]. All *P* values were two-tailed, and the significance level was set at *P* = 0.05. G*Power 3 was used to conduct the power calculation [39]. Plink software was employed to carry out the adjustment for age and gender factors in the genetic analysis [40].

## Supplementary Material

Supplementary Table 1

Supplementary Table 2
