# Supplementary material for: Genetic variants in RET, ARHGEF3 and CTNNAL1, and relevant interaction networks, contribute to the risk of Hirschsprung disease
Source: Aging (Albany NY). 2020 Mar 6;12(5):4379–93. doi: 10.18632/aging.102891 (PMC7093166; doi:10.18632/aging.102891)
Supplement: Supplementary Table 1 [file aging-12-102891-s002..docx]

| **Supplementary Table 1. Estimated haplotype frequencies and association significance .** | | | | | | | | | | | | | | | |  |  |  |  |
| --- | --- | --- | --- | --- | --- | --- | --- | --- | --- | --- | --- | --- | --- | --- | --- | --- | --- | --- | --- |
| Gene ID | Haplotype* | | | | | | | | | | |  |  |  | Haplotype frequency(%) | | X^2^ | *p* value | Odds Ratio (95%CI) |
| *RET* | rs2506030 | rs7069590 | rs2505998 | rs2435357 | rs752978 | rs74400468 | rs3026737 | rs1864402 | rs2075910 | rs2435353 | rs2075913 | rs17028 | rs2742240 | rs2435355 | HSCR | Control |  |  |  |
|  |  |  |  |  |  |  |  |  |  | C | A | C |  |  | 74.00(7.4) | 174.39(17.1) | 43.493 | **4.40 x 10^-11^** | 0.39(0.29-0.52) |
|  |  |  |  |  |  |  |  |  |  | C | T | C |  |  | 799.00(80.4) | 592.61(58.0) | 116.752 | **3.51 x 10^-27^** | 2.96(2.42-3.61) |
|  |  |  |  |  |  |  |  |  |  | T | A | T |  |  | 120.00(12.1) | 250.99(24.6) | 52.800 | **3.85 x 10^-13^** | 0.42(0.33-0.53) |
|  |  |  |  |  |  | G |  |  |  | C | A | C |  | T | 68.09(6.8) | 169.78(16.6) | 46.115 | **1.16 x 10^-11^** | 0.37(0.27-0.50) |
|  |  |  |  |  |  | G |  |  |  | T | A | T |  | C | 118.03(11.9) | 250.09(24.5) | 54.133 | **1.95 x 10^-13^** | 0.41(0.33-0.53) |
|  |  |  |  | C | C | G |  |  |  |  |  |  |  |  | 39.72(4.1) | 130.60(12.9) | 47.407 | **5.99 x 10^-12^** | 0.29(0.20-0.42) |
|  |  |  |  | C | T | G |  |  |  |  |  |  |  |  | 114.21(11.7) | 320.39(31.6) | 110.624 | **7.69 x 10^-26^** | 0.29(0.23-0.37) |
|  |  |  |  | T | C | G |  |  |  |  |  |  |  |  | 754.32(77.3) | 443.36(43.7) | 259.104 | **2.69 x 10^-58^** | 4.93(4.04-6.04) |
|  |  |  |  |  |  |  |  | G | A |  |  |  | T |  | 665.77(67.8) | 490.84(48.3) | 88.655 | **5.00 x 10^-21^** | 2.42(2.01-2.91) |
|  |  |  |  |  |  |  |  | G | G |  |  |  | A |  | 37.78(3.8) | 84.12(8.3) | 16.300 | **5.46 x 10^-5^** | 0.45(0.30-0.67) |
|  |  |  |  |  |  |  |  | T | G |  |  |  | A |  | 201.07(20.5) | 407.86(40.1) | 86.816 | **1.27 x 10^-20^** | 0.39(0.32-0.48) |
|  |  |  | A | T | C | G |  |  |  | C | T | C |  | T | 663.03(68.4) | 413.33(40.8) | 230.477 | **4.69 x 10^-52^** | 4.83(3.92-5.96) |
|  |  |  | G | C | T | C |  |  |  | C | T | C |  | T | 28.85(3.0) | 99.81(9.9) | 33.762 | **6.39 x 10^-9^** | 0.30(0.20-0.46) |
|  |  |  | G | C | T | G |  |  |  | T | A | T |  | C | 62.38(6.4) | 198.13(19.6) | 65.379 | **6.49 x 10^-16^** | 0.30(0.22-0.41) |
|  |  |  |  | C | T | C |  |  |  | C | T | C |  | T | 29.01(3.0) | 103.98(10.3) | 39.800 | **2.90 x 10^-10^** | 0.28(0.18-0.42) |
|  |  |  |  | C | T | G |  |  |  | C | A | C |  | T | 24.12(2.5) | 86.05(8.5) | 32.348 | **1.32 x 10^-8^** | 0.28(0.18-0.45) |
|  |  |  |  | C | T | G |  |  |  | T | A | T |  | C | 63.94(6.6) | 198.11(19.6) | 69.020 | **1.03 x 10^-16^** | 0.30(0.22-0.40) |
|  |  |  |  | T | C | G |  |  |  | C | T | C |  | T | 687.49(70.9) | 415.26(41.0) | 222.928 | **2.08 x 10^-50^** | 4.52(3.69-5.54) |
| *ARHGEF3* | rs11717604 | rs4681946 | rs11720618 | rs13070800 | rs11925835 | rs3732508 | rs9882898 | rs3732509 | rs3772219 | rs3732511 | rs1009119 | rs6978 | rs808 |  |  |  |  |  |  |
|  |  |  | G |  | C | G | G | C | A | G | C | A | A |  | 14.60(1.5) | 35.28(3.5) | 7.183 | **0.007** | 0.44(0.24-0.81) |
|  |  |  | G |  | C | G | G | G | C | C | T | G | A |  | 33.33(3.4) | 17.15(1.7) | 6.987 | **0.008** | 2.19(1.21-3.96) |
|  |  |  | G |  | T | A | A | G | A | C | T | G | A |  | 56.77(5.8) | 31.56(3.1) | 10.306 | **0.001** | 2.07(1.32-3.24) |
|  |  |  | G |  | T | G | A | G | A | G | C | A | A |  | 31.15(3.2) | 66.66(6.6) | 10.89 | **0.001** | 0.48(0.31-0.75) |
|  |  |  | C |  | C | G |  |  |  |  |  |  |  |  | 63.10(6.4) | 38.18(3.8) | 7.671 | **0.006** | 1.78(1.18-2.69) |
|  |  |  | G |  | T | G |  |  |  |  |  |  |  |  | 348.68(35.3) | 448.12(44.2) | 14.402 | **0.000** | 0.70(0.59-0.84) |
|  |  |  | G |  | C | A |  |  |  | G | C | A |  |  | 36.71(3.8) | 16.50(1.6) | 8.902 | **0.003** | 2.39(1.33-4.30) |
|  |  |  | G |  | T | G |  |  |  | C | T | G |  |  | 235.86(24.2) | 298.06(29.5) | 6.423 | **0.011** | 0.77(0.63-0.94) |
|  |  |  | G |  | C | A |  |  | A | C | T | G | A |  | 33.21(3.4) | 9.33(0.9) | 14.484 | **1.43 x 10^-4^** | 3.78(1.82-7.86) |
|  |  |  | G |  | C | A |  |  | A | G | C | A | A |  | 40.40(4.1) | 16.58(1.7) | 11.047 | **0.001** | 2.59(1.45-4.62) |
|  |  |  | G |  | T | G |  |  | A | C | T | G | A |  | 75.38(7.7) | 128.68(12.8) | 14.087 | **1.76 x 10^-4^** | 0.56(0.42-0.76) |
| *CTNNAL1* | rs10816766 | rs10979650 | rs4978766 | rs4978379 | rs2282206 | rs838816 | rs838817 | rs7021366 | rs7027874 | rs2289481 | rs2289480 |  |  |  |  |  |  |  |  |
|  | T | A | G | G | G | C | T | C | G | C | C |  |  |  | 128.44(13.1) | 107.72(10.8) | 4.145 | **0.042** | 1.33(1.01-1.75) |
|  |  | G | A | C |  |  |  |  |  |  |  |  |  |  | 775.37(77.7) | 866.99(85.0) | 3.453 | 0.063 | 0.80(0.62-1.01) |
|  |  | G | A | C |  |  |  | C |  |  |  |  |  |  | 486.58(49.0) | 583.93(57.4) | 6.785 | **0.009** | 0.79(0.66-0.94) |
|  |  | G | A | C |  |  |  | C | A | G | A |  |  |  | 463.91(47.0) | 552.81(54.4) | 5.074 | **0.024** | 0.81(0.68-0.97) |
|  |  | A | G | G |  | C |  | C |  |  |  |  |  |  | 131.65(13.3) | 112.57(11.1) | 4.394 | **0.036** | 1.33(1.02-1.75) |
|  |  | G | A | C |  | C |  | C |  |  |  |  |  |  | 6.43(0.6) | 37.61(3.7) | 19.731 | **9.04 x 10^-6^** | 0.18(0.08-0.42) |
|  |  | G | A | C |  |  |  | C | A |  |  |  |  |  | 465.53(47.2) | 553.44(54.5) | 4.852 | **0.028** | 0.82(0.68-0.98) |
|  |  | G | A | C |  |  |  | C |  | G |  |  |  |  | 472.94(47.6) | 554.62(54.5) | 4.789 | **0.029** | 0.82(0.68-0.98) |
|  | T | G | A | C |  |  |  | C |  |  |  |  |  |  | 290.55(29.3) | 364.32(36.0) | 4.562 | **0.033** | 0.81(0.67-0.98) |
| *Haplotypes were omitted from analysis if the estimated haplotype probabilities were less than 3%, CI = confidence interval, HSCR = Hirschsprung disease. | | | | | | | | | | | | | | | | | | | |
