# Supplementary material for: Genetic variants in RET, ARHGEF3 and CTNNAL1, and relevant interaction networks, contribute to the risk of Hirschsprung disease
Source: Aging (Albany NY). 2020 Mar 6;12(5):4379–93. doi: 10.18632/aging.102891 (PMC7093166; doi:10.18632/aging.102891)
Supplement: Supplementary Table 2 [file aging-12-102891-s001..docx]

| **Supplementary Table 2. Haplotype risks of *RET*, *ARHGEF3* and *CTNNAL1* in Hirschsprung disease** | | | | | | | | | | | |  |  |  |
| --- | --- | --- | --- | --- | --- | --- | --- | --- | --- | --- | --- | --- | --- | --- |
| Group | **Haplotype*** | | | | | | | | | | Haplotype frequency(%) | | Odds Ratio (95%CI) | *P* value |
|  | Risk Haplotype** | | | SNP (Risk/Non-risk allele) | | | | | | | HSCR | Control |  |  |
| *RET + ARHGEF3* | rs2506030 | rs7069590 | rs2435357 | rs11720618 (C/G) | rs11925835 (C/T) | rs3732508 (A/G) |  |  |  |  |  |  |  |  |
|  | G | T | T | C | C | G |  |  |  |  | 46.13(4.8) | 15.65(1.6) | 2.75(1.54-4.94) | **4.15 x 10^-4^** |
|  | G | T | T | C | T | G |  |  |  |  | 106.60(11.1) | 44.97(4.5) | 2.32(1.61-3.35) | **4.26 x 10^-6^** |
|  | G | T | T | G | C | A |  |  |  |  | 68.61(7.1) | 16.79(1.7) | 3.94(2.29-6.80) | **1.35 x 10^-7^** |
|  | G | T | T | G | C | G |  |  |  |  | 116.78(12.1) | 68.65(6.8) | 1.63(1.18-2.24) | **0.003** |
|  | G | T | T | G | T | A |  |  |  |  | 83.30(8.6) | 32.90(3.3) | 2.44(1.60-3.70) | **1.91 x 10^-5^** |
|  | G | T | T | G | T | G |  |  |  |  | 228.86(23.7) | 178.85(17.8) | 1.22(0.96-1.54) | 0.101 |
| *RET + CTNNAL1* | rs2506030 | rs7069590 | rs2435357 |  |  |  | rs10979650 (A/G) | rs4978766 (G/A) | rs4978379 (G/C) | rs7021366 (G/C) |  |  |  |  |
|  | G | T | T |  |  |  | A | G | G | C | 82.83(8.5) | 24.98(2.5) | 4.10(2.59-6.49) | **1.15 x 10^-10^** |
|  | G | T | T |  |  |  | G | A | C | G | 225.81(23.1) | 101.94(10.1) | 3.16(2.44-4.09) | **3.65 x 10^-19^** |
|  | G | T | T |  |  |  | G | A | C | C | 309.33(31.7) | 215.37(21.4) | 2.07(1.67-2.56) | **1.48 x 10^-11^** |
| *RET + ARHGEF3 + CTNNAL1* | rs2506030 | rs7069590 | rs2435357 | rs11720618 (C/G) | rs11925835 (C/T) | rs3732508 (A/G) | rs10979650 (A/G) | rs4978766 (G/A) | rs4978379 (G/C) | rs7021366 (G/C) |  |  |  |  |
|  | G | T | T | C | T | G | G | A | C | G | 31.25(3.2) | 8.40(0.8) | 3.92(1.81-8.50) | **2.05 x 10^-4^** |
|  | G | T | T | G | C | G | G | A | C | G | 51.88(5.4) | 26.41(2.6) | 2.09(1.28-3.40) | **0.003** |
|  | G | T | T | G | T | A | G | A | C | C | 63.32(6.6) | 16.42(1.6) | 4.31(2.47-7.55) | **3.85 x 10^-8^** |
|  | G | T | T | C | C | G | G | A | C | G | 29.02(3.0) | 6.98(0.7) | 4.38(1.90-10.11) | **1.76 x 10^-4^** |
|  | G | T | T | G | T | G | G | A | C | G | 66.18(6.9) | 41.17(4.1) | 1.71(1.13-2.59) | **0.010** |
|  | G | T | T | G | T | G | G | A | C | C | 118.31(12.3) | 108.30(10.8) | 1.13(0.84-1.52) | 0.433 |
| *Haplotypes were omitted from analysis if the estimated haplotype probabilities were less than 3%, **Risk haplotype within *RET* in European studies [23], CI = confidence interval, HSCR = Hirschsprung disease. | | | | | | | | | | | | | | |
